# Comparative study to evaluate dosimetric differences in patients of locally advanced carcinoma cervix undergoing intracavitary brachytherapy under two different anaesthesia techniques: an audit from a tertiary cancer centre in India

**DOI:** 10.1186/s43046-019-0003-2

**Published:** 2019-11-19

**Authors:** Divyesh Kumar, G. Y. Srinivasa, Ankita Gupta, Bhavana Rai, Arun S. Oinam, Pooja Bansal, Sushmita Ghoshal

**Affiliations:** 1 Department of Radiotherapy and Oncology, Regional Cancer Centre, Post Graduate Institute of Medical Education and Research, Chandigarh, India; 2 Department of Biostatistics, Post Graduate Institute of Medical Education and Research, Chandigarh, India

## Abstract

**Background:**

Carcinoma cervix is amongst the leading causes of mortality and morbidity in women population worldwide. High-dose-rate intracavitary brachytherapy (HDR-ICBT) post external beam radiation therapy (EBRT) is the standard of care in managing locally advanced stage cervical cancer patients. HDR-ICBT is generally performed under general anaesthesia (GA) in operation theatre (OT), but due to logistic reasons, sometimes, it becomes difficult to accommodate all patients under GA. Since prolonged overall treatment time (OTT) makes the results inferior, taking patients in day care setup under procedural sedation (PS) can be an effective alternative. In this audit, we tried to retrospectively analyse the dosimetric difference, if any, in patients who underwent ICBT at our centre, under either GA in OT or PS in day care.

**Results:**

Thirty five patients were analysed 16/35 (45.71%) patients underwent HDR-ICBT under GA while 19/35 (54.28%) patients under PS. In both groups, a statistically significant difference was observed between the dose received by 0.1 cc as well as 2 cc of rectum (*p* < 0.05), while the bladder and sigmoid colon had comparable dosages.

**Conclusion:**

Though our dosimetric analysis highlighted better rectal sparing in patients undergoing HDR-ICBT under GA when compared to patients under PS, PS can still be considered an effective alternative, especially in centres dealing with significant patient load. Further studies are required for firm conclusion.

## Background

Carcinoma cervix is a major health concern amongst the female population worldwide. In India, cervical cancer is the third most common cancer overall and second most common cancer in females [1]. The peak age of incidence of cervical cancer is 55–59 years, and the majority of patients report in the late stages of the disease [2]. Concurrent chemoradiation with high-dose-rate intracavitary brachytherapy (HDR-ICBT) is the standard of care in patients with locally advanced cervical cancer [3]. Brachytherapy can be delivered using either low dose rate (LDR) or high dose rate (HDR). HDR brachytherapy has largely replaced LDR brachytherapy due to its distinct advantages of small source and applicator size, short treatment times, and better control of source positioning and dose distribution. These factors favourably allow HDR brachytherapy to be delivered on an outpatient basis where in multiple fractions of brachytherapy can be administered simultaneously sandwiched during the course of external radiotherapy. For high-volume centres where large numbers of patients are treated, shorter treatment times with HDR brachytherapy allow multiple patients to undergo the brachytherapy procedure on the same day. The American Brachytherapy Society (ABS) recommends a cumulative external beam and intracavitary (EBRT+ICBT) radiation dose of approximately 80–90 Gy for definitive treatment of carcinoma cervix and that HDR brachytherapy to be performed under general anaesthesia (GA) [4]. In addition, the overall treatment time (OTT) of EBRT and brachytherapy should be less than 8 weeks, beyond which local control and survival has been shown to decrease by ~ 1% per day [5]. Although examination under anaesthesia (EUA) helps in better visualization of the diseased structure and delineation of parametrial extension of the disease, chances of anaesthesia-induced complications are more [6]. Moreover, a high burden of patients not only adds to the complexity of managing these patients under GA, but also increases the OTT.

The American College of Emergency Physicians (ACEP) defines procedural sedation (PS) as a technique of administering sedatives or dissociative agents with or without analgesics to induce a state that allows the patient to tolerate unpleasant procedures while maintaining cardiorespiratory function. It allows the patient to maintain oxygenation and airway control independently by depressing the level of consciousness [7].

At our institute, we routinely perform brachytherapy under GA. However, due to increased patient load, including referrals from other centres for brachytherapy, we recently have started performing HDR brachytherapy applications under PS in a day care setting in order to avoid increasing the OTT.

Since adequate muscle relaxation resulting in appropriate intracavitary application might be achieved under GA, we hypothesized that dosimetric outcome may vary between brachytherapy procedure done under GA in the operation theatre (OT) setup or PS done in a day care setting. Hence, an audit was carried out in the present study to compare and evaluate the dosimetric parameters in patients of carcinoma cervix who underwent HDR-ICBT under these two different setups with different anaesthesia.

## Methods

Dosimetric data of 35 patients with histopathologically proven locally advanced carcinoma cervix who had undergone HDR-ICBT from May 2018 to August 2018 at our centre were retrospectively analysed. All patients who had received EBRT of 46 Gy in 23 fractions using the 3-dimensional conformal radiotherapy (3DCRT) technique over four and a half weeks with concurrent chemotherapy (cisplatin 40 mg/m^2^) followed by HDR-ICBT of 9 Gy per session for 2 sessions (each application done at weekly intervals) as per the departmental protocol were included for analysis. The choice of the sedation technique was based on the local anatomy and the anticipated ease of application.

### Procedure under GA

After pre-anaesthesia clearance and routine preparation, patients who were taken up for ICBT in major OT under GA were asked to lie down in lithotomy position. After adequate cleaning and draping, Foley’s catheter was inserted, and the balloon was inflated with 7 cc of normal saline. Size and extent of disease and local anatomy were assessed by EUA which included per-speculum, per-vaginal, and per-rectal examinations. After assessment of uterine length and angle, and adequate dilation of the cervical os, the most suitable central tandem was inserted into the uterus. The ovoids were placed in the right and left vaginal fornices equidistant from the central tandem. The vagina was packed with roller gauze to displace the bladder further anteriorly and the rectum posteriorly to minimize the dose to these organs and to immobilize the applicators.

### Procedural sedation

The same pre-procedural protocol was followed for patients taken up for ICBT in day care minor OT setup under PS. Intravenous pentazocine (30 mg) and intravenous promethazine (25 mg) intravenously were given for PS.

### Post application procedure

Post application, patients were taken up for planning computerized tomography (CT) scan in the dedicated departmental CT scan machine (GE Healthcare Technologies, Wankesha, WI, USA). Subsequently, the patients’ bladder was filled with 2 ml of iohexol diluted with 18 ml of normal saline, and planning CT images were acquired with the patient in supine position and applicator in situ using multi-slice CT scanner with slice thickness of 2.5 mm. The images were then transferred to Eclipse treatment planning system (v.8.6, Varian Associates, Palo, Alto, CA, USA); organs at risk (OARs), i.e. bladder, rectum and sigmoid, were contoured according to the Groupe Européan de Curiethérapie - European Society for Radiotherapy & Oncology (GEC-ESTRO) guidelines. A dose of 9 Gy HDR was prescribed to point A as per departmental protocol (dwell positions for both ovoids are 3,4,5,6 while central tandem positions are 1,3,5,7,9,12,15,18), and the plan was evaluated. The optimisation was done when required with the aim of delivering a minimum dose of 100% to the HRCTV (high-risk clinical target volume).

### Dosimetry

**Fig. 1:**
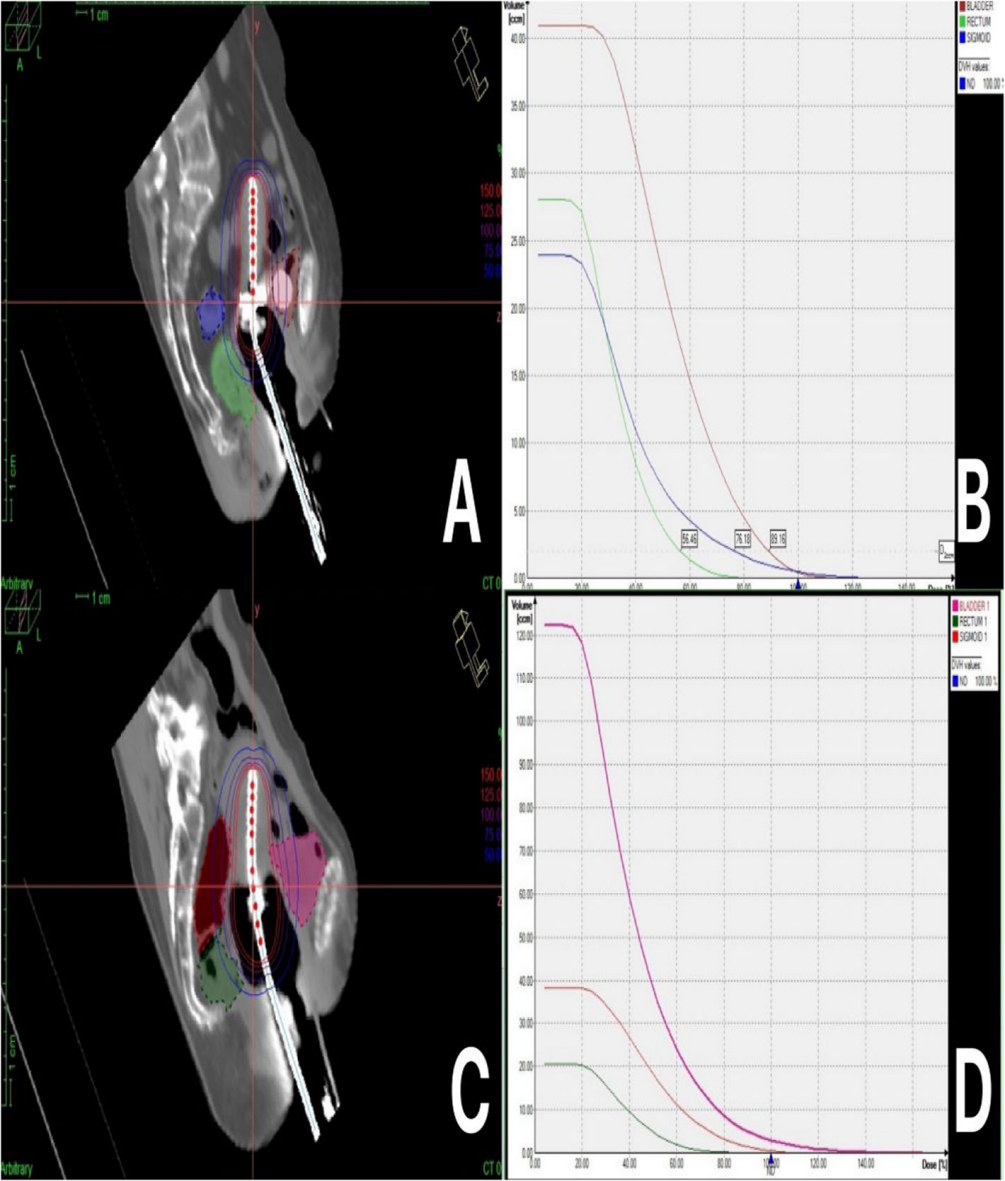
(**a–d**) Isodose distribution and DVH parameters for OARs in a patient treated under procedural sedation (**a, b**) and under general anaesthesia (**c, d**)

Generated dose volume histograms (DVH) were analysed, and EQD2 (dose equivalent of 2 Gy) doses received by 0.1 cc and 2 cc of organs at risk (OARs), i.e. bladder, rectum and sigmoid colon, were evaluated (Fig. 1). EQD2 dosage was calculated by combining both EBRT dose and dosage received during HDR-ICBT.

### Statistical analysis

SPSS 22.0 (SPSS Inc., Chicago, IL) software was used for data analysis. Standard methods of descriptive statistics (arithmetic mean with the standard deviation and the numerical range from minimum to maximum value) were used for 0.1 cc and 2 cc dosage, respectively (Tables 2 and 3). Statistical significance of differences amongst the examined groups (GA and PS) was tested using two sample independent t-test at 5% level of significance. *p* value < 0.05 was considered as significant.

## Results

### Patient and treatment characteristics (Table 1)

The number of patients in the GA and PS groups was 16 and 19, respectively. Median age of patients in the GA group was 53 years, and in the PS group 49 years. The most common presenting symptoms were bleeding per vaginum and discharge per vaginum in both groups. The majority of patients in both the groups were of stage IIB. All patients underwent EBRT to a total dose of 46 Gy in 23 fractions with concurrent cisplatin (40 mg/m^2^) over four and a half weeks followed by 2 sessions of ICBT (9Gy HDR per session) with a gap of 1 week between the sessions.

**Table 1:** Patient demographic characteristics

Attributes	GA	PS
Median age (years)	53	49
Stage		
I B2	2	2
II A2	1	2
II B	11	12
III B	2	3
ICRT		
Dose per fraction (Gy)	9	9
Median ovoid size	Medium	Medium
Median tandem length (cm)	5	5

### Comparison of EQD2 doses received by 0.1 cc of OARs (Table 2)

The mean dose received by the sigmoid colon was 75.43 Gy (range 52.28–122.12 Gy) and 74.91 Gy (range 53.13–98.84 Gy) of point A (*p* = 0.924), while the mean dose received by the bladder was 105.16 Gy (range 63.65–127.09 Gy) and 100.23 Gy (range 60.4–172.49 Gy) in the GA group and PS group, respectively (*p* = 0.537). The rectum received a mean dose of 72.60 Gy (range 55.14–88.30 Gy) under GA and 83.60 Gy (range 60.58–1118.62 Gy) in the PS group (*p* < 0.05).

**Table 2:** EQD2 dose received by 0.1 cc OARs

Organs at risk	Group	*N*	Mean	Std. deviation	Min	Max	*p* value	95% CI of the difference	
								Lower	Upper
Sigmoid colon	GA	16	75.4331	18.30633	52.28	122.12	.924	− 10.37846	11.40576
	PS	19	74.9195	13.30924	53.13	98.84			
Bladder	GA	16	105.1681	17.58910	63.65	127.09	.537	− 11.14112	21.00263
	PS	19	100.2374	27.12735	60.4	172.49			
Rectum	GA	16	72.6000	9.23701	55.14	88.30	.009*	− 19.07379	− 2.92621
	PS	19	83.6000	13.40413	60.58	118.62			

* *p*-values are significant at 5% level of significance

### Comparison of EQD2 doses received by 2 cc of OARs (Table 3)

The mean dose received by 2 cc of sigmoid colon was 62.20 Gy (range 49.68–77.05Gy) and 62.91 Gy (range 50.99–79.74 Gy) of point A dose (*p* = 0.789); mean dose received by the bladder was 79.51 Gy (range 59.18–95.62 Gy) and 77.96 Gy (range 56.64–96.24 Gy) in GA and PS groups, respectively (*p* = 0.677). The rectum received a mean dose of 63.47 Gy (range 54.86–77.48 Gy) and 69.54 Gy (range 54.21–90.95 Gy) in GA and PS groups, respectively (*p* < 0.05).

**Table 3:** EQD2 dose received by 2 cc OARs

Organs at risk	Group	*N*	Mean	Std. deviation	Min	Max	*p* value	95% CI of the difference	
								Lower	Upper
Sigmoid Colon	GA	16	62.2031	8.33009	49.68	77.05	.789	− 6.04624	4.62828
	PS	19	62.9121	7.19461	50.99	79.74			
Bladder	GA	16	79.5156	10.70180	51.18	95.62	.677	− 5.94628	9.03964
	PS	19	77.9689	10.97947	56.64	96.24			
Rectum	GA	16	63.4744	5.78111	54.86	77.48	.024*	− 11.27340	− .86417
	PS	19	69.5432	8.73826	54.21	90.95			

* *p*-values are significant at 5% level of significance

There was no significant difference in the dose received by 0.1 cc and 2 cc of sigmoid colon and bladder amongst both the groups. However, a significant difference was observed between the dose received by 0.1 cc as well as 2 cc of rectum (*p* < 0.05) in both the groups.

## Discussion

ICBT along with EBRT is the cornerstone of curative treatment in locally advanced carcinoma cervix. The ease of applicator placement makes HDR-ICBT treatment convenient to be used even in an out-patient setting. In the present audit comparing GA and PS, we observed no significant difference in dose received by 0.1 cc and 2 cc of sigmoid colon and bladder amongst both the groups. However, a significantly higher dose was received by 0.1 cc as well as 2 cc of rectum under PS compared to GA (*p* value < 0.05). This could be due to the better adequate vaginal packing as a result of better muscle relaxation under GA. A study of comparison of HDR-ICBT dosimetry with and without anaesthesia done by Sharma et al. showed that mean dose to the bladder reference point was 5.03 Gy (71.85% of point A dose) in the anaesthesia group compared to 4.90 Gy (70% of point A dose) in patients without anaesthesia (*p* value 0.6) and mean dose to the rectal point was significantly higher in anaesthesia group compared to patients without anaesthesia (5.09 Gy v/s 4.49 Gy, *p* value 0.01) [8]. In a similar study done by Rathore et al., mean dose to the bladder was in the range of 17.7–69.3% and 15.54–74.24% in anaesthesia and conscious sedation (CS) groups respectively, mean dose to the rectum was 32.5–77.73% and 21.07–79.16% in the anaesthesia and CS groups respectively, and they concluded that dosimetric parameters in both the groups were similar and did not depend on the type of anaesthesia [9]. Both these studies used 2D conventional planning for dosimetric evaluation; we on the contrary used CT-based volumetric planning for dosimetric evaluation of the OARs.

Various anaesthetic forms, depending on the comfort of the patient, have been recommended byABS [10]. A study done by Shirakawa et al. showed that caudal epidural anaesthesia is an effective and safe anaesthesia option during HDR-ICBT for carcinoma cervix [11]. Chen et al. in their study showed that local vaginal anaesthesia with 10% lidocaine solution can significantly decrease the degree of painful sensation during HDR-ICBT and is safe to administer for the procedure for cervical cancer [12]. Study done by Leong et al. concluded that outpatient combined intracavitary and interstitial brachytherapy for cervix cancer with sedation and local anaesthesia is feasible and safe and could potentially lead to significant cost savings [13].

In a study evaluating complications associated with the usage of different anaesthetic techniques during HDR brachytherapy in patients of carcinoma cervix, Lim et al. concluded that GA had significantly more complications than topical anaesthesia or CS (both *p* < 0.001) [14]. This may be of particular concern where the patients have to be exposed to anaesthesia multiple times during repeated brachytherapy sessions. Though in our study we did not aimed to evaluate anaesthesia-related complications, no associated complications were reported.

In a non-dosimetric study done by Bhanabhai et al., the effectiveness of CS for pain control during HDR-ICBT using a ring-and-tandem applicator system was evaluated and it was demonstrated that good pain control could be achieved with CS [15]. In view of the retrospective nature of our study, pain relief and patient comfort was not assessed.

There are certain limitations of our study. Foremost are its retrospective design and a small patient sample size. Secondly, pain relief and patient comfort were not assessed because of retrospective nature of analysis. Also, there was an inherent selection bias in choosing patients for ICBT procedure as only those patients who were anatomically suitable and in whom easy applicator placement was anticipated were chosen for ICBT under PS.

## Conclusion

Though, as per our analysis, ICBT done under GA results in reduced rectal doses when compared to that performed under PS, the limitations of our study restricts us from drawing a firm conclusion. Nevertheless, PS can still be considered as a more convenient, cost-saving and less complicating alternative in centres where large numbers of patients are treated and/or performing multiple brachytherapy applications, especially in low-/middle-income countries. Further prospective studies are required to validate our results.

## Data Availability

Records from the Department of Radiotherapy, PGIMER, Chandigarh, India.

## Abbreviations

EBRT: External beam radiotherapy
EQD2: Dose equivalent of 2 Gy
EUA: Examination under anaesthesia
GA: General anaesthesia
HDR: High dose rate
ICBT: Intracavitary brachytherapy
LDR: Low dose rate
OTT: Overall treatment time
PS: Procedural sedation
